# Seaweed and yeast extracts as sustainable phytostimulant to boost secondary metabolism of apricot fruits

**DOI:** 10.3389/fpls.2024.1455156

**Published:** 2025-01-24

**Authors:** Noemi Gatti, Moez Maghrebi, Graziella Serio, Carla Gentile, Victor V. Bunea, Ivano Vigliante, Camille Boitte, Christian Garabello, Valeria Contartese, Cinzia M. Bertea, Giuseppe Mannino

**Affiliations:** ^1^ Department of Life Sciences and Systems Biology, Plant Physiology Unit, University of Turin, Turin, Italy; ^2^ Department of Biological, Chemical and Pharmaceutical Sciences and Technologies (STEBICEF), University of Palermo, Palermo, Italy; ^3^ Research and Development Department, Green Has Italia S.p.A, Canale, CN, Italy; ^4^ Ecole de Biologie Industrielle, University of Paris-Seine, Cergy, France

**Keywords:** biostimulant, polyphenols, secondary metabolites, radical scavenging activity, HPLC-DAD-MS/MS, *Prunus armeniaca*

## Abstract

In our study, we investigated the effects of Expando, a commercial biostimulant derived from seaweed and yeast extracts, on the secondary metabolism of Lady cot and Orange prima apricot cultivars. Notably, treatments with or 5.0 L/ha of Expando improved fruit uniformity and harvests synchronization, providing agronomic benefits. Expando positively influenced the biosynthesis of essential bioactive compounds such as polyphenols, flavonoids, proanthocyanidins, and anthocyanins in both apricot pulp and peel, as validated by HPLC-ESI-MS/MS analysis. These metabolic enhancements translated into significantly increased total antioxidant activity, particularly evident in the peel samples. Principal Component Analysis (PCA) revealed distinct effects of the 5.0 and 4.0 L/ha treatments, distinguishing them from lower doses and the control group. Our findings emphasize the potential of Expando to enhance the phytochemical profile of apricot fruits, positioning biostimulants as pivotal tools for improving fruit quality and sustainability in agriculture. Expando offers a sustainable and eco-friendly approach to enhancing crop yield and nutritional value, representing a significant step towards more resilient and environmentally conscious farming practices. Further research is needed to explore its broader implications and optimize application strategies for commercial orchards.

## 1 Introduction

The growing global population and concomitant increase in food demand necessitate the adoption of sustainable agricultural practices to ensure food security while minimizing environmental impact (Canellas et al., 2015). Although the situation has evolved slightly, the core needs persist, and in some cases, the current condition may have further deteriorated, with no immediate resolution in view (Nadathur et al., 2024). Consequently, conventional agricultural practices, which have historically played a significant role in boosting food production, are now recognized for their substantial environmental consequences. Indeed, reliance on chemical fertilizers and pesticides has resulted in soil degradation, water pollution, and a decline in biodiversity (Wesseler, 2022). For instance, soil degradation, characterized by the loss of soil fertility, structure, and organic matter, poses a significant threat to long-term agricultural productivity. Additionally, the leaching of chemical inputs into waterways has led to water pollution, adversely affecting aquatic life and contaminating drinking water supplies. In addition to the implications due to both acute (toxicity at field application doses) and chronic (toxicity at consumer doses) exposures (Dhankhar and Kumar, 2023), the widespread use of chemical formulation has also been linked in the decline of beneficial insect populations, including pollinators like bees, which play a critical role in crop productivity. Furthermore, the loss of biodiversity, both above and below ground, disrupts ecosystem functions necessary for sustainable agriculture, such as natural pest control and nutrient cycling.

In addressing these challenges, biostimulants have emerged as a promising solution to enhance crop productivity and resilience in an environmentally sustainable manner. In the biological sense, a biostimulant refers to substances or microorganisms that enhance plant growth and health by stimulating natural processes, such as nutrient uptake and stress resilience. Conversely, as a regulatory category (EC1107/2009 and EU2019/1009), biostimulants are defined by legal frameworks that distinguish them from traditional fertilizers and pesticides, focusing on their intended purpose to improve plant performance without specific nutrient content (Garg et al., 2024). This dual meaning underscores the importance of both their physiological role in agriculture and the need for regulatory standards to ensure their efficacy and safety in horticultural practices.

Biostimulants encompass a diverse array of products, including seaweed extracts, humic acids, protein hydrolysates, and beneficial microbes, that function by activating natural processes in plants and soils (Rana et al., 2023). Unlike conventional fertilizers that primarily supply nutrients, biostimulants enhance nutrient use efficiency, improve plant metabolism, and increase the plants’ ability to withstand abiotic stresses such as drought, salinity, and extreme temperatures (Wszelaczyńska et al., 2019).

In this context, the utilization of seaweed and yeast extract as biostimulants has garnered considerable attention due to their beneficial biological effects on crop performances (Ali et al., 2021; Ashour et al., 2021; Cozzolino et al., 2021; Cristofano et al., 2021; Khan et al., 2009; Rana et al., 2023). Seaweeds, abundant in coastal regions, and yeast extracts, derived from microbial fermentation, serve as valuable sources of bioactive compounds that exert beneficial effects on plants. Among these, extracts from *Ascophyllum nodosum* and yeast for bakery fermentation stand out as specific examples with distinct advantages. *Ascophyllum nodosum*, a brown seaweed commonly found along the coasts of the North Atlantic, is rich in bioactive compounds such as alginates, mannitol, and various micronutrients, which have been shown to enhance plant growth, improve stress resistance, and promote root development (Campobenedetto et al., 2021; El-Nakhel et al., 2022; Taskos et al., 2019). According to the White Paper by the European Biostimulants Industry Council (EBIC), seaweed extracts play a significant role as biostimulants by promoting plant growth and enhancing resilience to stress through the activation of specific biochemical pathways, such as those involving hormones and polysaccharides, which contribute to improved nutrient uptake and root development (Gupta et al., 2023). Similarly, yeast extracts sourced from bakery fermentation, particularly those rich in nucleotides and amino acids, can stimulate plant metabolism and enhance overall vigor, leading to improved nutrient uptake and increased resilience to environmental stressors (Dima et al., 2023, Dima et al., 2020; Shahrajabian et al., 2023). Together, these specific types of extracts can be effectively utilized to optimize plant health and productivity in agricultural settings.

*Prunus armeniaca* L., commonly known as apricot, holds a significant place in European agriculture and culinary culture. Recognized for its sweet fruits, apricot tree prospers in various European regions with suitable climates, including Mediterranean countries like Spain, Italy, and Greece, as well as Central European nations such as France and Hungary. Apricots are appreciated for their flavor, vibrant colors, and nutritional values, making them a subject of interest for scientific inquiry regarding their phytochemical composition, health benefits, and culinary applications within the European context (Mohd Wani et al., 2017). For instance, apricots are rich in essential vitamins, minerals, and antioxidants, known for their contribution to a balanced and healthy diet. They are particularly valued for their high content of vitamin A and C, potassium, and dietary fiber, which promote overall health and well-being.

The aim of this work is to investigate the potential of a biostimulant based on *A. nodosum* and yeast extract in improving the yield, quality, and functional properties of *Prunus armeniaca* L. (apricot) trees, which rank among the most important fruit trees worldwide. Biostimulants have demonstrated an elicitor effect on annual plants, increasing the production of secondary metabolites with functional and defense properties, thus reducing the reliance on pesticides and enhancing the health-promoting properties of plant-derived products (Mrid et al., 2021). However, research on the use of biostimulants, particularly on perennial fruit trees like apricots, face limitations such as the need for extensive experimental areas and the lengthy juvenile phase before fruit production. In order to fill this gap, the potential derived from the application of the formulation was evaluated on two apricot cultivars, “Lady Cot” and “Orange Prima.” The selected cultivars were chosen for their economic significance and prevalence in the local agricultural landscape. By focusing on varieties that are widely cultivated, we aim to ensure that our findings are not only relevant but also applicable to the current agricultural practices of the region. Each cultivar possesses distinct characteristics and consumer preference, which make them suitable candidates for evaluating the effects of biostimulants. Furthermore, the trial sites were selected to capture a diverse range of local growing conditions, encompassing variations in soil types, climate, and farming practices. This strategic selection allows us to comprehensively assess the biostimulant effectiveness across different environmental scenarios. The evaluation covered both edible tissues (pulp and peel) of the fruits, with focus on quantifying bioactive compounds such as polyphenols, flavonoids, flavan-3-ols, and anthocyanins, as well as assessing the functional properties, including radical scavenging and reducing activity. Through this research, our goal is to provide valuable insights into the potential of Expando to enhance the nutritional quality and health-promoting properties of apricot fruits, thereby contributing to the advancement of sustainable and high-quality fruit production practices.

## 2 Materials and methods

### 2.1 Chemicals and reagents

All reagents and chemicals used in this study were of analytical grade, selected to ensure high purity and reliability of experimental results. Ethanol used as solvent for fruit extraction, was purchased from Merck Life Science (Milan, Italy) with purity ≥ 99.5%. For the spectrophotometric assays, Folin-Ciocalteu reagent, sodium carbonate, aluminum chloride, 4-dimethylaminocinnamaldehyde, sodium acetate, chloride acid and formic acids, were purchased from VWR International (Milan Italy). For the evaluation of antioxidant properties, Trolox, gallic acids, 2,4,6-tris(2-pyridyl)-s-triazine (TPTZ), ferric chloride, 2,2’-azino-bis acid (ABTS) and 2,2-diphenyl-1-picrylhydrazyl (DPPH) radicals for the respective scavenging assays were obtained from Thermo Fisher Scientific Inc. (Monza, Italy). For HPLC analysis, solvents were HPLC grade and included methanol, acetonitrile, formic acid (as mobile phase modifier). Authentic reference standards for polyphenols were provided at high purity (> 98%). Both standard and solvent were provided by VWR International (Milan, Italy). Milli-Q water, used as the main solvent, was produced with a Millipore filtration system (Millipore Corporation, Burlington, Massachusetts, USA). All reagents were stored under controlled conditions, following the manufacturers’ guidelines, to maintain stability and integrity throughout the study.

### 2.2 Experimental field trial

**Figure 1:**
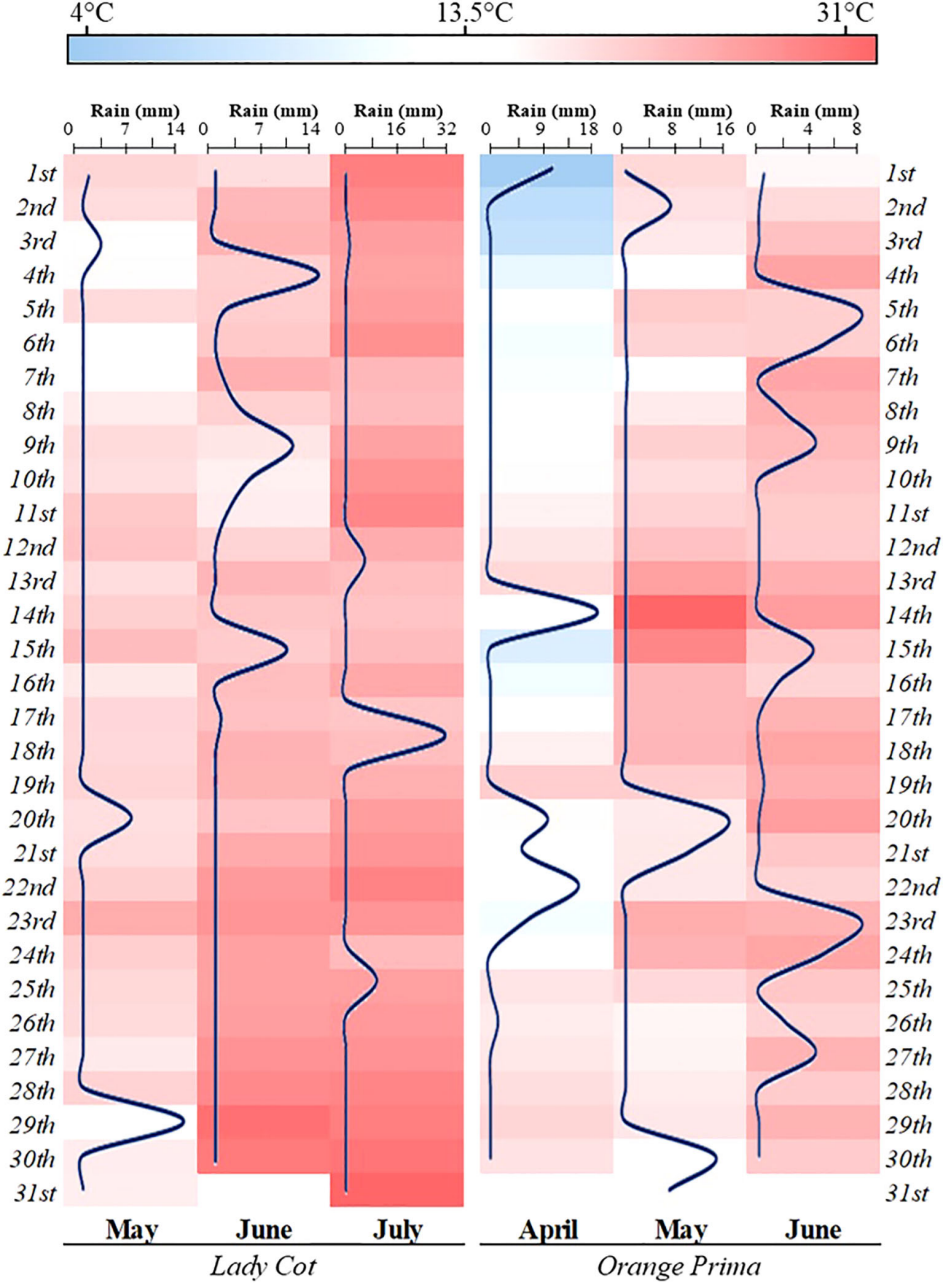
Heatmap showing the climatic conditions recorded during the field trials at the two experimental stations. The gradation from blue to red indicates temperature changes, expressed in degrees Celsius (°C). Additionally, a blue line on the map represents the amount of precipitation, measured in millimeters (mm).

The experiments were carried out in 2020 at two different sites in Italy: Santarcangelo di Romagna (Rimini, Emilia Romagna by AGRICOLA 2000 S.C.p.A) and Canosa di Puglia (Barletta-Andria-Trani, Puglia by AgroService R&S S.r.l). The first site (44°05’64’’ N, 12°42’90’’ E, 41 m altitude) experiences a Mediterranean climate with an average yearly temperature of 18.3°C. The peak monthly temperature occurs in July (20-29°C), and the lowest in January (0-8°C). According to the meteorological station of Rimini (distance 6.2 Km from the experimental field) the site receives 470.95 mm of annual precipitation, most of which falls in September, with around 30% of the annual total occurring between April and July. The average relative humidity is 77%. During the study, the climatic conditions recorded by meteorological stations were illustrated in **Figure 1**. In this location, *Prunus armeniaca* (cv. Lady cot) trees were planted in 2016 at a density of 555 trees per ha, with rows spaced 6.0 m apart. Each plot measured 90.0 m² (6.0 m × 15.0 m) and contained 5 plants. The soil was classified as clay loam, comprising 35.4% sand, 29.5% silt, 1.80% organic matter, and 33.3% clay, with a pH of 7.95 and a cation exchange capacity (CEC) of 35.23. The second site (41°08’26’’ N, 15°59’37’’ E, 146 m altitude) has a temperate climate, with an average annual temperature of 17.4°C. The warmest month is August (20-31°C), while the coldest is January (4-11°C). According to the meteorological station of La Palombe, Andria (distance 8.86 Km from the experimental field), annual rainfall is 535.69 mm, peaking in December, and approximately 35% of the total rainfall occurs between April and July. The average relative humidity at this site is 65%. The climatic conditions for the experimental period are also shown in **Figure 1**. Here, *Prunus armeniaca* L. (cv. Orange prima) trees were planted in 2012 at a density of 500 trees per hectare, with a row spacing of 5.0 m. The plots measured 60.0 m² (5.0 m × 12.0 m) and contained 6 plants. The soil, also Clay Loam, was composed of 37.3% sand, 30.5% silt, 1.79% organic matter, and 32.2% clay, with a pH of 7.45 and a CEC of 34.67.

### 2.3 Biostimulant formulation and trial design

The same biostimulant formulation and experimental protocol were applied in both trial fields. A commercial biostimulant (Expando, Green Has Italia S.P.A., Canale, Italy) was used for treatments, applied via foliar application using a back-spray sprayer with a water volume of 1000L/ha. This method ensured uniform spraying over the leaf surfaces. According to the product label, the biostimulant contains *A. nodosum* and bakery yeast extract, 3% (w/w) organic nitrogen, 4% (w/w) phosphoric anhydride, 6% (w/w) potassium oxide, 0.02% (w/w) boron, 0.1% (w/w) molybdenum, 0.02% (w/w) manganese, and 12% (w/w) organic carbon. Moreover, the dry matter of the biostimulant represent the 45% (g/L) of the solution. The pH of the product in a 1% (w/w) water solution is 6.50 ± 0.50, and its electrical conductivity in a 1g/L water solution is 350 μS cm^−1^.

According to our previous researches on tomato and peaches (Mannino et al., 2020a; Mannino et al., 2020b), the experiments included four treatments where the biostimulant was applied at different concentrations (5.0 L/ha, 4.0 L/ha, or 2.5 L/ha) either when the fruits reached about 40% of their final size or at the onset of fruit coloration. Water-sprayed plants served as controls. The treatments were arranged in a randomized complete block design with four blocks, each containing 30 (6 × 5) unit plots. The spacing between adjacent blocks and plots was 6 m and 3 m, respectively. Treatments were randomly assigned to plots within each block, with separate randomization for each block.

All the fruits that were considered ripe based on coloration and established criteria (Bolat and Ikinci, 2020) were harvested during three different stages (at least 200 fruits per each harvesting time) corresponding to the progressive ripening of the apricot varieties under investigation. To avoid drift effects, only the three middle plants in each plot were sampled. Data on fruit number, average fruit weight, and total yield per plot were recorded. Immediately after harvesting, the fruits were quickly frozen at -80°C for further analysis.

### 2.4 Preparation of fruit extracts

To prepare the extracts, the harvested fruits were first separated into five groups (each composed from 15 fruits). Then, the peel was removed from the pulp and both parts were crushed and homogenized separately using a high-speed homogenizer to ensure uniform consistency. For each extraction, 1 gram of whole homogenized peel or pulp was accurately weighed and transferred into a clean tube containing 10 mL of 70% (v/v) ethanol. The mixture was briefly vortexed to ensure initial mixing and then subjected to sonication (20 kHz; 100 watts) for 30 minutes to enhance the extraction efficiency of bioactive compounds. Following sonication, the samples were stirred continuously at 6000 g for 48 hours at room temperature to facilitate thorough extraction. After the mixing period, the mixtures were centrifuged at 8000 g for 10 minutes to separate the solid residues from the liquid extracts. To ensure the exhaustiveness of the extraction process, the same procedure was repeated twice for both peel and pulp samples. The resulting supernatants were carefully transferred into new tubes and stored at -20°C until further analysis. This extraction procedure was performed in triplicate for both peel and pulp to ensure reproducibility and reliability of the results. Each step was conducted under controlled conditions (in the dark and at 20°C) to maintain the integrity of the bioactive compounds in the extracts from both the peel and pulp. Extracts prepared according this methodology was used for analysis as described in section 2.4 and section 2.6.

### 2.5 Determination of bioactive compounds via UV/Vis assay

#### 2.5.1 Total polyphenol content

Total Phenolic Content (TPC) was determined using the Folin-Ciocalteu assay, which quantifies phenolic compounds through a redox reaction. This reaction involves the oxidation of phenols in an alkaline solution by the yellow molybdotungstophosphoric heteropolyanion reagent, resulting in the formation of a blue molybdotungstophosphate complex that can be measured calorimetrically at 725 nm (Verica et al., 2009). Briefly, 20 µL of each sample extract was added to 20 µL di Folin reagent and 10 µL of 20% (w/V) sodium carbonate and water up to 200 µL. After heating 1h at 80°C the absorbance was read using UV-Probe 1280 Spectrophotometer (Shimadzu, Italy). The results were expressed as mg of gallic acid equivalent (GAE) per 100 g of fresh weight (FW). All measurements were performed in triplicate to ensure accuracy and reproducibility.

#### 2.5.2 Total flavonoid content

The quantification of total flavonoid content (TFC) was performed using Aluminium Chloride Assay, as previously reported (Alajil et al., 2021). Briefly, 20 μL of 5% (w/V) sodium nitrite was added to 20 μL of sample, and after thorough vortexing the tubes were incubated at room temperature for 5 minutes before adding 20 μL of 10% (w/V) aluminium chloride and incubating for an additional 6 minutes. Finally, 40 μL of 4% (w/V) sodium hydroxide was added, the contents are diluted to 200 μL with distilled water, and after allowing the mixture to develop for 15 minutes, the absorbance at 510 nm is measured against the blank. An external calibration curve of rutin, ranging from 1 mg/mL to 7.81×10^-^³ mg/mL, was used to quantify the flavonoids in the extracts. The results were expressed as Rutin Equivalent (RE) per 100 g of FW. All measurements were performed in triplicate to ensure the accuracy and reproducibility of the results.

#### 2.5.3 Total proanthocyanidin content

The total proanthocyanidin content (TPAC) was assessed using the DMAC (4-dimethylaminocinnamaldehyde) assay, a reliable method for proanthocyanidin (PAC) quantification, leading a measurable color change at 640 nm (Mannino et al., 2021). Briefly, 20 μL of sample extract was incubated to 180 μL 1% (w/V) DMAC solubilized in 75% (v/v) ethanol acidified with 0.5 (v/v) hydrochloric acid. After 20 min of incubation at room temperature, the absorbance was monitored at 640 nm. For quantification, PAC-A2 solution was used, with dilutions ranging from 0.1 mg/mL to 3.125×10^-^³ mg/mL. Results were expressed as mg of PAC-A2 type equivalent (PACE) per 100 g of FW. Triplicate measurements ensured result reliability and consistency.

#### 2.5.4 Total anthocyanin content

The total anthocyanin content (TAC) was determined using the differential pH method, a spectrophotometric technique enabling the estimation of anthocyanins based on their reversible structural changes in different pH environments (Pisani et al., 2021). Briefly, 150 μL of sample extract was added to 350 μL of buffer solution of potassium chloride (pH 1.0) or sodium acetate (pH 4.5). After 5 min incubation, the absorbance of each solution is measured using a spectrophotometer at two 510 nm (which corresponds to the maximum absorbance of anthocyanins) and 700 nm (used to correct for any background color). Data were expressed as mg of Cyanidin Equivalent (CE) per 100 g of FW. Triplicate measurements ensured result reliability and consistency.

### ​2.6. Determination of secondary metabolites

The determination of the phytochemical profile of apricot hydroalcoholic extracts was conducted using a High-performance liquid chromatography (HPLC) system, following established protocols (Mannino et al., 2022). The setup comprised a liquid chromatography (LC) system (Agilent Technologies 1200, Santa Clara, California, United States) equipped with a diode array detector (DAD) and an ion trap mass spectrometry (MS) system (Agilent Technologies 6300, Santa Clara, California, United States) featuring an electrospray ionization (ESI) source (Agilent Technologies 1200, Santa Clara, California, United States). Chromatographic separation was achieved using a C18 Luna reversed-phase column (3.00 μm, 150.00 × 3.0 mm i.d.) maintained at 25°C by a thermostat module (Agilent Technologies 1200, Santa Clara, California, United States), with a constant flow rate of 0.2 mL min^-^¹. The UV-Vis spectra of eluted compounds were recorded within the range of 220 nm to 800 nm. Mass spectrometry analysis was conducted in positive mode for anthocyanins and in negative mode for other polyphenolic compounds. The nitrogen flow rate was set at 15.0 mL min^-^¹, and the flow temperature was maintained at 350°C. Capillary voltage was set at ± 1.5 kV. Compound identification was achieved by comparing the retention time (RT), UV-Vis spectra, and mass fragmentations of eluted compounds with those of authentic reference compounds sourced from Sigma-Aldrich, St. Louis, Missouri, USA. For polyphenol compounds, the elution process employed a multistep linear solvent gradient, commencing with an initial solvent B concentration of 15% (v/v) and progressing to 45% (v/v) over 15 minutes. Subsequently, the gradient was increased to 70% (v/v) B over 20 minutes. At the conclusion of each run, the initial solvent concentration was restored and maintained for an additional 10 minutes before the subsequent injection. The sample injection volume was 10 μL. For the analysis of anthocyanidin compounds, a binary solvent system was employed, consisting of MilliQ H_2_O acidified with 10% (v/v) formic acid (Sigma-Aldrich, St. Louis, Missouri, USA) (Solvent A) and 50% (v/v) MetOH acidified with 10% (v/v) formic acid (Solvent B). At the conclusion of each run, the initial solvent concentration was reinstated and sustained for an additional 10 minutes before the subsequent injection. Each sample injection volume was 5 μL. All analyses were conducted in triplicate to ensure accuracy and reliability.

### 2.7 Determination of antioxidant activity

#### 2.7.1 Reducing capacity

The Ferric Reducing Antioxidant Power (FRAP) colorimetric method was employed to evaluate the reducing ability of apricot fruit extracts. This assay relies on the capacity of an antioxidant compound to reduce ferric ions (Fe^3+^) to ferrous ions (Fe^2+^), forming a blue complex (Fe^2+^/TPTZ) with increased absorption at 593 nm (Mannino et al., 2020b). Briefly, 20 µL of the apricot extract was mixed with the FRAP solution, which consists of a mixture of 300 mM acetate buffer, 10 mM TPTZ, and 20 mM ferric chloride solution (8:1:1 v/v/v). The mixture was incubated at 37°C for 30 minutes to allow for the reduction reaction to occur fully. After incubation, the absorbance of the resulting blue solution was measured at 593 nm using a spectrophotometer. The results were expressed as Trolox Equivalent (TE) per 100 g of fresh weight. All measurements were conducted in triplicate to ensure consistency and accuracy of the results.

#### 2.7.2 Radical scavenging activity

The antioxidant activities of apricot fruit extracts were evaluated using two common methods, namely the ABTS assay and the DPPH assay (Rumpf et al., 2023). For the ABTS assay, 7 mM ABTS solution and 2.45 mM potassium persulfate were incubated 16 h before performing the determinations, allowing it to stand in the dark at room temperature. Prior to the assay, the ABTS solution was diluted with phosphate-buffered saline (PBS) to an absorbance of 0.70 at 734 nm. Different amount of apricot extract was then mixed with the ABTS solution, and the decrease in absorbance was measured after a 6-minute incubation at room temperature. In the DPPH assay, a stable DPPH radical solution was prepared by dissolving DPPH in methanol to a concentration of 0.1 mM. A set volume of apricot extract was added to the DPPH solution and mixed thoroughly. The mixture was allowed to incubate in the dark for 30 minutes, after which the absorbance was measured at 517 nm. Results from both assay were expressed as mmol Trolox Equivalent (TE) per 100 g of FW.

### 2.8 Statistical analysis

The data obtained from all experiments were presented as mean values ± standard deviation (SD) of three independent replicates. Statistical analysis was conducted using SPSS software (version 27). Significant differences among the various treatments were determined using one-way analysis of variance (ANOVA), followed by Tukey’s Honestly Significant Difference (HSD) test. Data from metabolomics analysis were normalized to the median, followed by a log base 10 transformation. The data were then scaled by mean-centering and divided by the square root of the standard deviation of each variable (Pareto scaling). Baselining was performed by subtracting the average value of each variable across the dataset, ensuring that the analysis focused on treatment-induced variations. The effects of data normalization, transformation, and scaling are illustrated in **Supplementary Figure S1**. For volcano plot, fold change threshold of 1.5x, corresponding to a log2 fold change of approximately ±0.585, to highlight significant differences in metabolite levels between treatments. Additionally, we set a p-value threshold of 0.05, which is commonly used in scientific research to determine statistical significance. To ensure clarity and enhance the interpretability of our results, we applied a logarithmic transformation to our data using a log2 scale. Orthogonal Partial Least Squares Discriminant Analysis (OPLS-DA) to evaluate the effects of biostimulant treatments on the metabolic profiles of apricot fruits. To validate our OPLS-DA model and assess its predictive capability, we conducted Cross-Validated Analysis of Variance (CV-ANOVA) calculating Accuracy, Determination Coefficient (R^2^) for both X- and Y-axis, and Cross-Validated R^2^ (Q^2^). Volcano and OPLS-DA analysis were performed using the online tool Metabo-Analyst v 6.0. Due the limited number of comparisons, no multiple testing correction was employed.

## 3 Results and discussion

### 3.1 Maturation time is affected by biostimulant application

The production yield, maturity, and quality of field-grown fruits can be affected by many environmental stressors. These factors have the potential to decrease both the agronomic yield and the quality of fruit (Lamichhane et al., 2018). Several scientific evidences have demonstrated that biostimulant formulations applied during fruit ripening can influence several plant physiological pathways, leading to improvement in production yield (Castiglione et al., 2021; Matthews et al., 2022).

In general, seaweed and yeast-based biostimulants are gaining considerable interest in agricultural production systems due to their bioactive components that result in beneficial effects on crop production. Although extraction methods can significantly affect the yield and concentration of bioactive compounds obtained from these raw materials, these substances inherently possess phytostimulant properties that can enhance plant performance (Ali et al., 2021; Dulanlebit and Hernani, 2023). However, the demonstrated effects seems to be influenced by the type of plant to which the formulation is administered (El Boukhari et al., 2020).

**Figure 2:**
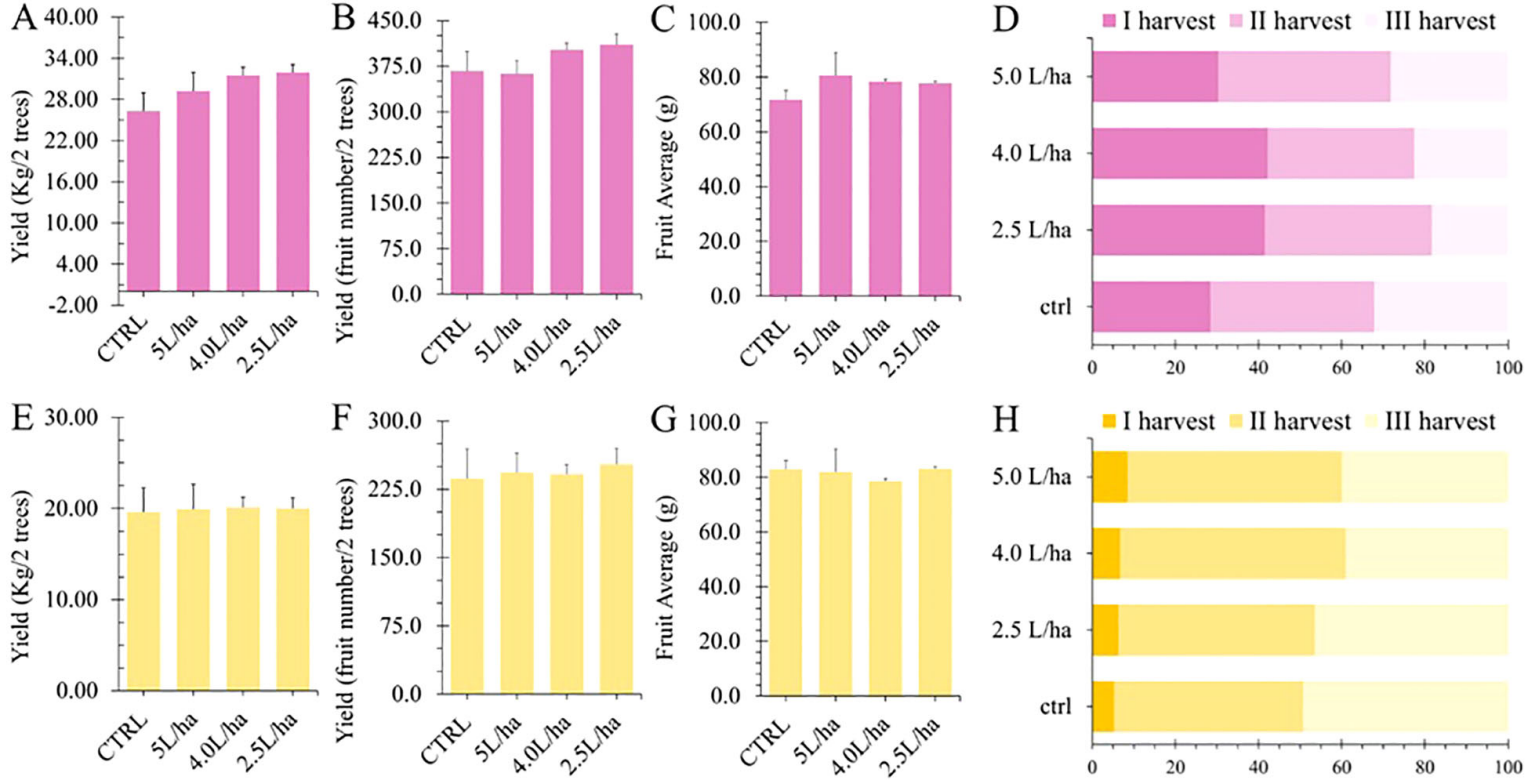
Agronomic yield data recorded for Lady cot **(A–D)** and Orange prima **(E–G)** varieties. **(A, E)** show the production weight per two trees. **(B, F)** report the number of produced fruits per two trees. **(C, G)** report Fruit Weight Average expressed as gram. **(D, H)** show the percentage of fruit collected at different harvesting time.

In this study, we evaluated the impact of applying a commercial seaweed extract in combination with yeast extract in order to monitor productivity performances and other functional properties. However, under our experimental conditions and against our expectations, the biostimulant treatments did not statistically (*p* < 0.05) affect the fruit yield of both varieties, either in terms of number of harvestable fruits or total weight (**Figures 2A, B, E, F**). Moreover, there was no statistical impact on the average fruit weight (**Figures 2C, G**). Biostimulants based on algae extracts are well known in the market for their ability to increase fruit size through several mechanisms, including upregulation of growth hormones such as auxins and gibberellins (Ali et al., 2021). In addition, these extracts can influence the activity of aquaporins, which improve water uptake and distribution in cells, thus contributing to increased fruit turgor and size (Sujata et al., 2023). However, while there was no observed increase in the number of fruits (**Figures 2B, F**), plants treated with the higher dosages (4.0 or 5.0 L/ha) demonstrated the ability to produce a more uniform number of harvestable fruits in lower timing. Indeed, most of the fruits were harvested during the first harvesting time, since they had met the predetermined maturity criteria (**Figures 2D, H**). Fruits that mature simultaneously are crucial for mechanical harvesting, as this synchrony ensures efficient collection and minimizes the risk of damage to the fruit and the harvesting equipment. By aligning the maturation timing, growers can optimize labor costs and enhance overall yield, making the harvesting process more effective and economically viable.

These results are partially in agreement to those obtained in our previous work in 2020, where we evaluated the effects of applying the same biostimulant on *Solanum lycopersicum* (var. Micro-Tom) fruits. Although the application protocol was the same, there were variations in experimental designs in terms of growth conditions. Indeed, tomatoes were grown with irrigation, humidity, light exposure, and temperature totally controlled by an automated system within a greenhouse. In this case, an increase in total production yield (+110%), a decrease in fruit ripening time (about two weeks), and a significant increase in fruit size (+85%) was observed (Mannino et al., 2020a). On the other hand, these results appear also in agreement with the findings of Tarantino et al. (2018), who, in their assessment of the effects of three different commercial biostimulants (HENDOPHYT, RADICON, and ERGOSTIM) on *Prunus armenica* trees (apricot) during two consecutive seasons, noted an acceleration in fruit ripening without a corresponding improvement in agricultural yield (Tarantino et al., 2018). A recent study determining the efficacy of seaweed extract treatments as an organic alternative to CPPU treatments for improving kiwifruit development, production, and quality demonstrates similar results. The research revealed that the application of seaweed extract (Agrogain) altered the double sigmoidal growth curve of kiwifruit during fruit development. Specifically, the SWE treatment, at 3000 ppm applied 10 days after fruit set, significantly enhanced various biochemical parameters, including soluble solids content, titratable acidity, sugars, and ascorbic acid levels, outperforming the CPPU-treated fruits. In terms of harvest timing, fruits treated with seaweed extract were ready for harvest 3–6 days earlier than the control group, while CPPU-treated fruits were harvested 8 days ahead of the control. Furthermore, seaweed extract-treated fruits exhibited better storage performance, including the lowest percentage of physiological weight loss and a superior SSC acid ratio. The post-harvest life of seaweed extract-treated fruits was longer than that of CPPU-treated fruits, resulting in an extended shelf life for kiwifruit (Rana et al., 2023).

The result shown in this work, might suggest that Expando is able to influence fruit maturation by reducing the ripening time, thereby leading to a more homogeneous production. This hypothesis aligns with the research conducted by Chouliaras et al. (2009), who tested a biostimulant based on *A. nodosum* extracts on olive trees. Although the authors did not observe significant effects on total yield following the application of the formulation, they recorded an acceleration of fruit ripening in accordance with olive color change (Chouliaras et al., 2009). Similarly, the same authors evaluated the foliar application of the same biostimulant on kiwifruits, highlighting a remarkable effect on fruit size and maturation by 10–15 days (Chouliaras et al., 1997). Furthermore, other works using commercial seaweed extracts obtained from *A. nodosum* have reported similar trends in Clementine mandarins, Navelina oranges (Fornes et al., 2002), and berry grapes (Khan et al., 2012; Sabir et al., 2014; Taskos et al., 2019).

### 3.2 HPLC-DAD-MS/MS analysis showed a different regulation of secondary metabolites in apricot fruits following biostimulant treatment

Metabolic analysis plays a key role in elucidating the intricate changes in metabolism that can occur following the application of a treatment. By delving into the complex network of metabolic pathways, this analytical approach provides a detailed understanding into how treatments influence cellular and systemic biochemical processes (Mena et al., 2016).

Here, we conducted targeted metabolic analysis to identify the main phenolic compounds content in apricot fruits. Our HPLC-DAD-MS/MS analyses reported the presence of different compounds belonging to six chemical classes. In particular, nine were flavonols [Kaempferol-3-O-hexoside isomer (#7), Kaempferol-3-O-glucuronide (#8), Kaempferol-3-O-hexoside isomer2 (#9), Kaempferol-3-O-rhamnoside (#11), Quercetin-3-O-rutinoside (#19), Quercetin-3-O-hexoside isomer2 (#21), Quercetin-3-O-hexoside isomer (#22), Quercetin (#27), Kaempferol (#29)], eight were flavanones [Naringenin-7-O-rutinoside (#3), Naringenin-7-O-hexoside isomer (#13), Naringenin-7-O-glucuronide (#14), Eriodictyol 7-O-rutinoside (#17), Eriodictyol 7-O-neohesperidoside (#20), Eriodictyol 7-O-hexoside isomer (#31), Eriodictyol (#32), Naringenin (#33)], five flavanonols [Dihydromyricetin-3-O-hexoside isomer (#1), Dihydroquercetin-3-O-hexoside isomer2 (#23), Dihydromyricetin (#24), Dihydroquercetin-3-O-hexoside isomer (#25), Dihydrokaempferol (#30)], four were flavonones [Luteolin-7-O-rhamnoside (#6), Luteolin-7-O-glucuronide (#10), Luteolin 7-O-Rutinoside (#18), Luteolin (#28)], four were proanthocyanidins (PACs) [PAC-A type were dimer (#4), PAC-B type dimer (#12), PAC-A type trimer (#35), three were flavan-3-ols [Catechin (#2), Epicatechin (#5) and Catechin 3’,5-dihexoside isomer (#15)], two were 4’-methoxy-flavanones (Hesperetin (#16), Hesperetin-7-O-rutinoside (#26)] and a O-methylated-flavonol [Isorhamnetin-3-O-rutinoside (#34)] (**Supplementary Table S1**).

**Figure 3:**
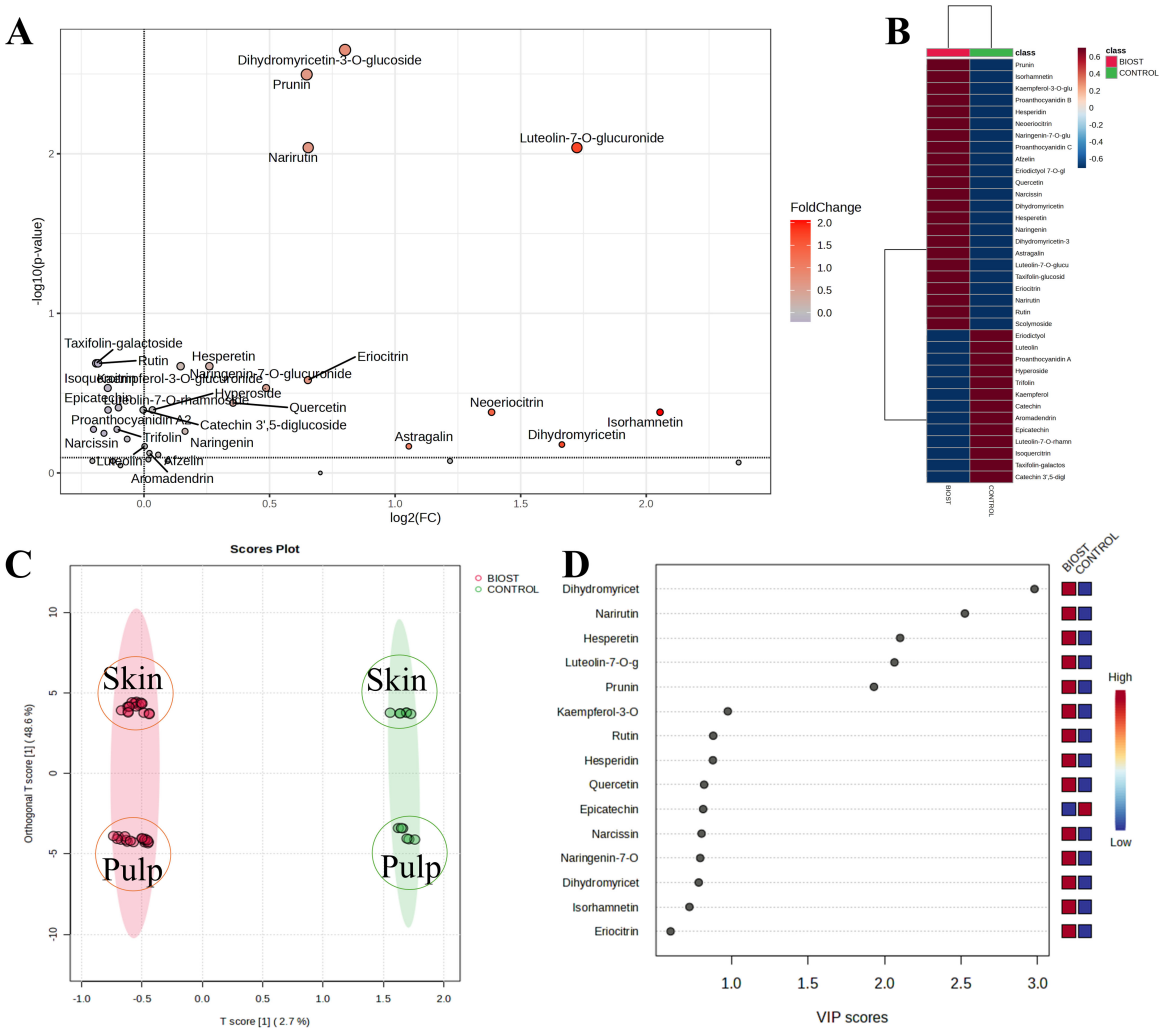
Metabolic analyses performed on apricot extracts following the application of Expando (BIOST) or simply water (CONTROL). **(A)** shows the Volcano plot combining the results of Fold Change (FC) analysis and T-tests into a single plot, allowing significant features to be distinguished based on their biological and statistical significance. **(B)** shows the heatmap visualizes all the variables analyzed, showing how they are affected in the two experimental groups (BIOST and CONTROL). **(C)** depicts the Orthogonal Partial Least Squares Discriminant Analysis (OPLS-DA), representing the average treatment effect and within-treatment variation, describing the residual systematic variation unrelated to the treatment. For *o1*, R^2^X: 0.486, R^2^Y: 0.038; Q^2^: 0.327. For *p1*, R^2^X: 0.027, R^2^Y: 0.818; Q^2^: 0.415. **(D)** shows the VIP plot reporting the important features expressed as VIP scores, indicating the main variables that differentiate between BIOST and CONTROL on Component 1.

To determine if treatment with the biostimulant could induce changes in the secondary metabolism of the analyzed fruits, quantitative data were compiled into a single database. This database included potential variables such as genotype (OP: Orange prima; LC: Lady cot), tissue type (P: pulp; S: skin), treatment application (CTRL: no treatment; BIOST: treatment with Expando), and different treatment concentrations (5.0 L/ha; 4.0 L/ha; 2.5 L/ha; 0 L/ha). Given that the compounds were quantitatively distributed differently across the two genotype and tissues analyzed, the data were first normalized to the median values, transformed to Log10, and then scaled using the Pareto method. This approach facilitated a more linear and representative data distribution, effectively minimizing potential false negatives. Results from normalization process are included in **Supplementary Figure S1**. The normalized data were then analyzed with biostimulant application as the sole variable. In this initial visualization, the effects of genotype, tissue type, and different biostimulant concentrations were neutralized. This approach aimed to determine if there were genuine shifts in secondary metabolism due to the biostimulant treatment (**Figure 3**). Consequently, regardless of the aforementioned variables, the volcano plot generated as log_2_(BIOST/CTRL) revealed that certain metabolites remained unchanged. These unchanged metabolites included Eriodictyol-hexoside isomer, PAB-B1, Taxifolin-hexoside isomer, and Eriodictyol (**Figure 3A**). The remaining compounds were differently regulated following application of the biostimulant (**Figure 3B**). In particular, the compounds that generated the most significant p-values were dihydromyricetin-hexoside isomer, prunin, narirutin, and luteolin-7-hexoside isomer. Using this same dataset, an Orthogonal PLS-DA (OPLS-DA) was performed. Despite the significant impact of tissue composition on Component 2, as evidenced by the distinct distribution of skins and pulps within the confidence regions of the respective control (CTRL) and biostimulant-treated (BIOST) groups, a clear separation between the two experimental groups was achieved (**Figure 3C** **).** This initial analysis suggested that were indeed a measurable effect of the biostimulant application on the analyzed fruits. The compounds that underwent the most significant metabolic regulation are listed in **Figure 3D**, while the metabolites that most discriminate between tissues (Pulp or Peel) are shown in **Supplementary Figure S2**.

**Figure 4:**
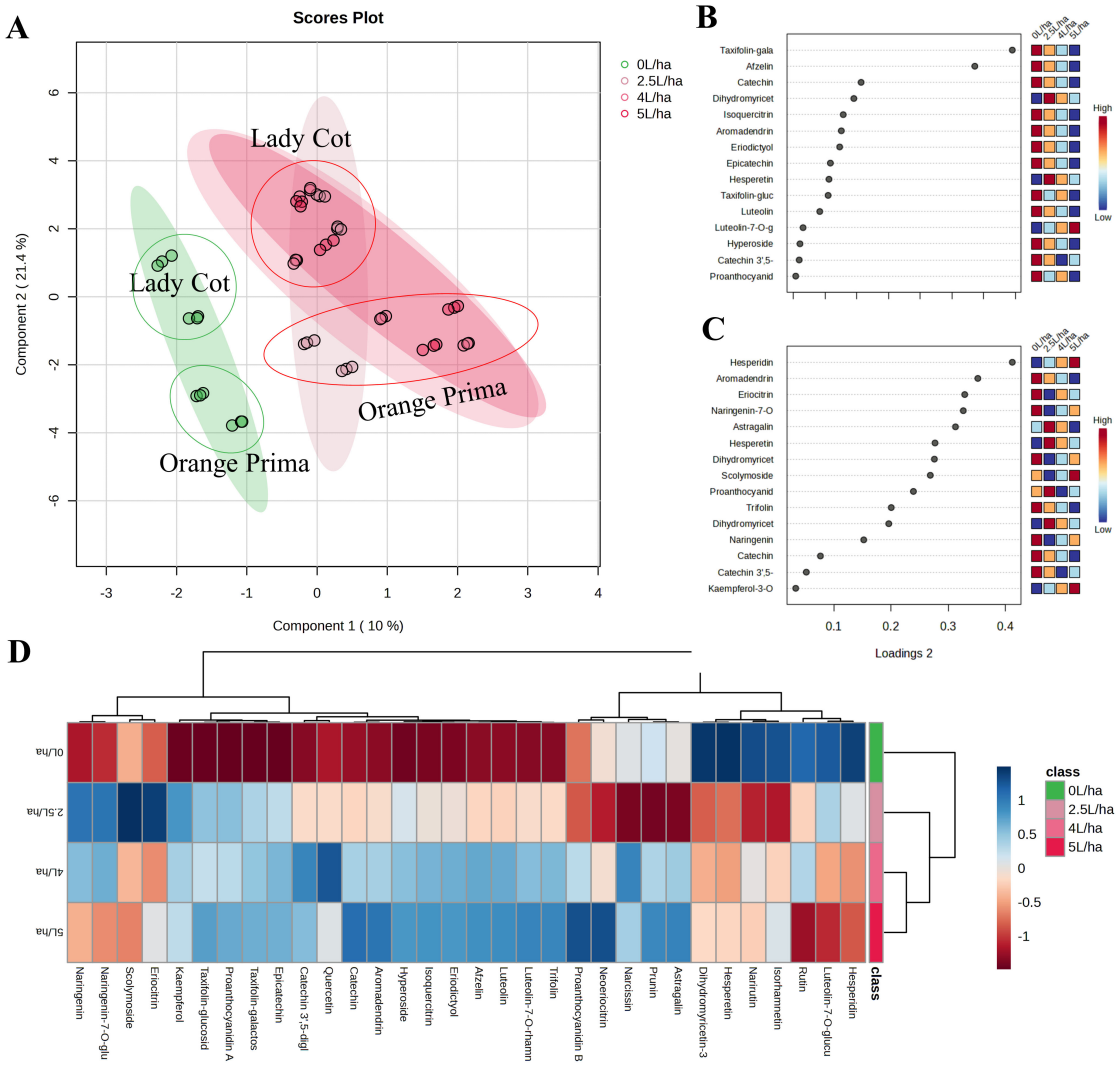
Metabolic analyses performed on apricot extracts following the application of different concentration of Expando (5.0 L/ha, 4.0 L/ha or 2.5 L/ha) or simply water (0 L/ha). **(A)** depicts the Sparse Partial Least Squares Discriminant Analysis (SPLS-DA), representing the average treatment effect and within-treatment variation, describing the residual systematic variation unrelated to the treatment. **(B, C)** show the VIP plot reporting the important features expressed as VIP scores, indicating the main variables that differentiate the different treatment on Component 1 or Component 2, respectively. **(D)** shows the heatmap visualization coupled with Hierarchical Clustering Dendrogram, showing how the different metabolites affected the different experimental groups.

As a second approach, an additional variable was included in order to understand whether different concentrations of the biostimulant used could actually induce different effects than the control (**Figure 4**). Using the same approach, a sparse PLS-DA (SPLS-DA) was then originated (**Figure 4A**). The distribution of samples on the SPLS-DA showed that untreated *Prunus armeniaca* trees produced fruit with a markedly different metabolic profile from trees that were instead treated with Expando at the different concentrations tested (**Figure 4A**). This figure agrees with the results shown in **Figure 3C**. However, the uneven distribution across different treatments (5.0, 4.0, or 2.5 L/ha) suggested that dosage has minimal influence on metabolic variations. The effects observed at the highest concentration are likely detectable even at the lowest concentration of the biostimulant. Component 1 predominantly discriminates the biostimulant effect, separating CONTROL samples (0 L/ha) from other effective dosages with positive values. Component 2, however, minimally influences the changes attributed to different concentrations resulting from biostimulant application and seems to be more discriminatory towards genotype. Positive Component 1 values tend to discriminate the Lady cot variety, while negative values tend to discriminate the Orange prima variety. The key metabolites enabling discrimination between CONTROL and different concentrations of BIOST (2.5, 4.0, or 5.0 L/ha) are listed in **Figure 4B**, while those that allow discrimination between the two genotypes (Lady cot or Orange prima) are given in **Figure 4C**. In contrast, the general effect of applying different concentrations of the biostimulant on apricot fruit is depicted in **Figure 4D**. The heatmap visualization demonstrates clear changes in plant secondary metabolism following treatment with the biostimulant. Hierarchical clustering dendrogram combined with heatmap visualization confirms the initial hypothesis. Specifically, fruits harvested from trees treated with water alone showed significantly lower levels of the analyzed compounds compared to those treated with the biostimulant. Although this effect does not lead to significant discrimination between treatments in SPLS-DA, individual differences allowed for the separation of the highest doses (5.0 and 4.0 L/ha) from the lowest (2.5 L/ha) (**Figure 4D**). However, it is worth noting that the strongest influence on the phytochemical profile appears to be the inherent differences in cultivar composition, rather than the biostimulant treatment itself. This is not unexpected, as the role of a biostimulant is not to drastically alter the phytochemical profile, which could compromise the traceability and originality of the product. Instead, its function is to enhance the existing phytochemical content while preserving the natural composition of the cultivar. Consequently, the biostimulant induces notable changes that support the fruit’s natural metabolic processes without transforming them.

The application of biostimulants such as Expando may affect specific biochemical pathways in apricot fruit, particularly those involved in the biosynthesis of secondary metabolites. Based on the targeted metabolic analysis conducted in our study, it could be possible that treatment with biostimulants increases the activity of key enzymes in the phenylpropanoid pathway, including phenylalanine ammonia lyase (PAL), chalcone synthase (CHS) and flavanone 3-hydroxylase (F3H) (Mannino et al., 2022). In addition, application of the biostimulant could stimulate proanthocyanidin synthesis by promoting the accumulation of these compounds through the activation of enzymes such as leucoanthocyanidin dioxygenase (LDOX), which catalyzes the formation of complex flavonoids (Mannino et al., 2022). An avenue to validate this hypothesis is through targeted transcriptional analysis, which would allow gene expression of enzymes involved in the metabolic pathways of interest to be monitored. This analysis could reveal significant changes in the levels of specific transcripts in response to biostimulant application, providing direct evidence on the activation of the proposed biochemical pathways and contributing to a deeper understanding of the molecular mechanisms underlying the observed metabolic changes.

By enhancing the plant’s metabolic performance, the biostimulant leads to increased levels of existing metabolites, aligning with the principle that biostimulants optimize intrinsic biological processes rather than triggering a complete reorganization of the fruit’s metabolic landscape.

### 3.3 Functional properties of apricot fruits are diversely influenced by the treatment with the biostimulant

Today, the improvement of crop cultivation technologies is mainly focused on enhancing crop productivity, yield quality and simultaneously mitigating environmental risks. The use of biostimulants is actually under investigation for its potential to not only increase plant productivity and resistance against biotic and abiotic stresses but also to enhance nutritional and functional properties of plant edible parts (Cocetta and Ferrante, 2020; Fan and Critchley, 2024). Functional properties are generally assessed through quantitative and qualitative analyses aimed at finding variation in phytochemical profiles after biostimulant treatments (Kocira and Kocira, 2019; Szparaga et al., 2018).

The term “biostimulant” occupies a gray area in terms of regulation, and it’s important to clarify its role. According to current EU regulatory frameworks (EC1107/2009 and EU2019/1009), biostimulants cannot legally claim to replace fertilizers or pesticides. Biostimulants are defined as products that enhance plant nutrient use efficiency, tolerance to abiotic stress, or crop quality, without providing essential nutrients or directly controlling pests. However, the challenge arises because some biostimulants can indirectly influence plant growth or stress resistance, leading to effects that may resemble those of fertilizers or pesticides. For example, a biostimulant might improve root health and nutrient uptake, which could be perceived as replacing a fertilizer, or it could boost a plant’s natural defenses, indirectly reducing pest damage. This ambiguity makes the regulation of biostimulants particularly complex. While they play a distinct role, their overlapping effects with fertilizers and pesticides raise important questions about labeling and marketing. The EU2019/1009 regulatory focus is on ensuring that biostimulants are clearly distinguished by their mode of action, emphasizing support for plant health and growth without making claims that would classify them as traditional agricultural inputs like fertilizers or pesticides (Fan and Critchley, 2024).

**Figure 5:**
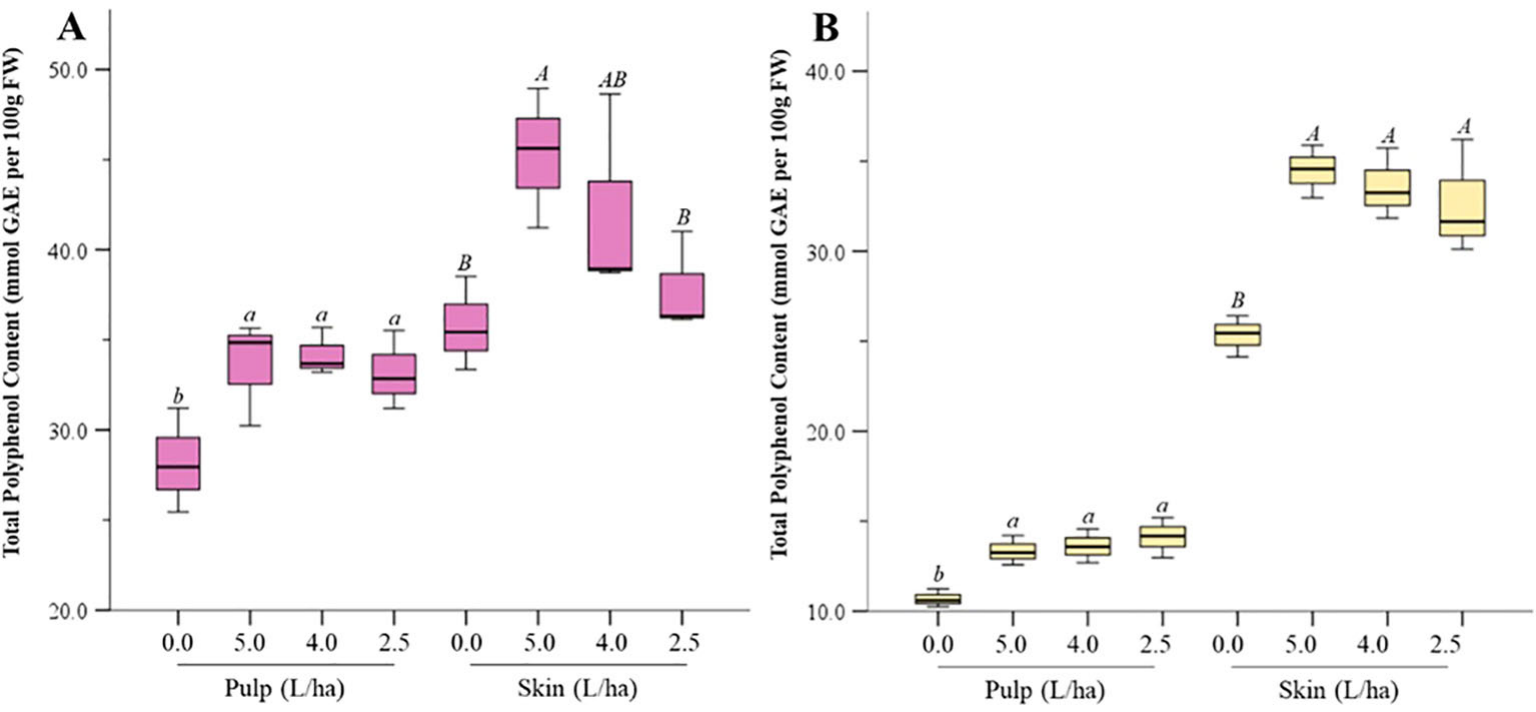
Total polyphenol content (TPC) measured in the pulp or skin of apricot harvested from Lady cot **(A)** or Orange prima **(B)** treated with different dosages of biostimulants (5.0 L/ha, 4.0 L/ha or 2.5 L/ha) or with water alone (0.0). Values are expressed as mmol of Gallic Acid Equivalent (GAE) per 100 g of fresh weight (FW). Within each box, horizontal black lines indicate median values, while boxes extend from the 25th to the 75th percentile of the distribution of values in each group. Moreover, the extended vertical lines indicate the standard deviations. For each panel, within the same variety (Lady cot or Orange prima) different lowercase (pulp) or uppercase (skin) letters indicate significant differences at p ≤ 0.05, as measured by Tukey’s multiple interval test.

In our experiments, treatment with the biostimulant increased the total phenolic content (TPC) of apricot fruits significantly (p ≤ 0.05) in both pulp and peel (**Figures 5A, B**). For instance, in Lady cot, the 5.0 L/ha treatment increased TPC levels in peel by 1.26 folds and in pulp by 1.10 folds compared to the control. Similar increases were observed in Orange prima cv., with the 5.0 L/ha treatment resulting in a 1.11-fold increase in pulp and a 1.24-fold increase in peel. Generally, all treatments increased TPC levels, with Lady cot showing a dose-dependent response, unlike Orange prima. The significance of these findings to the consumer lies primarily in the potential health benefits associated with increased levels of TPC in fruits. Phenolic compounds are well-known for their antioxidant properties, which contribute to improved nutritional quality and may offer protective effects against certain diseases. In this case, the biostimulant treatments, particularly at 5.0 L/ha, led to measurable increases in TPC in both the peel and pulp of Lady Cot and Orange Prima apricot cultivars, enhancing the fruit’s potential health benefits. Polyphenols, found abundantly in plant-based foods, have garnered attention for their beneficial effects on health [Cory et al., 2018; Pandey and Rizvi, 2009; Lorenzo et al., 2021]. They play a role in improving lipid profiles, blood pressure, insulin resistance, and reducing systemic inflammation (Cory et al., 2018; Lorenzo et al., 2021; Pandey and Rizvi, 2009). Moreover, polyphenols affect the gut microbiome, leading to better human health (Rana et al., 2022). Moreover, it’s important to consider that while the biostimulant improves TPC levels, the overall effects on taste, texture, and shelf-life, which are significant to consumers, were not the focus of this study. Future research will focus on the nutritional profile of the treated fruits, with particular attention to factors related to consumer consumption and health benefits. Additionally, the dose-dependent response observed in Lady Cot but not in Orange Prima highlights that biostimulants may interact differently with each cultivar, suggesting that consumer benefits might vary depending on the fruit variety and specific growing conditions.

While biostimulant treatments have generally been observed to increase total phenolic content (TPC) across a variety of fruit-producing species, such as Italian apples, sweet peppers, certain tomato cultivars, and red grapevine varieties (Graziani et al., 2020; Klokić et al., 2020; Parađiković et al., 2011), it is important to note that not all biostimulants are expected to yield identical results. The effectiveness of a biostimulant depends on its specific composition, the plant species or cultivar, and environmental factors.

**Figure 6:**
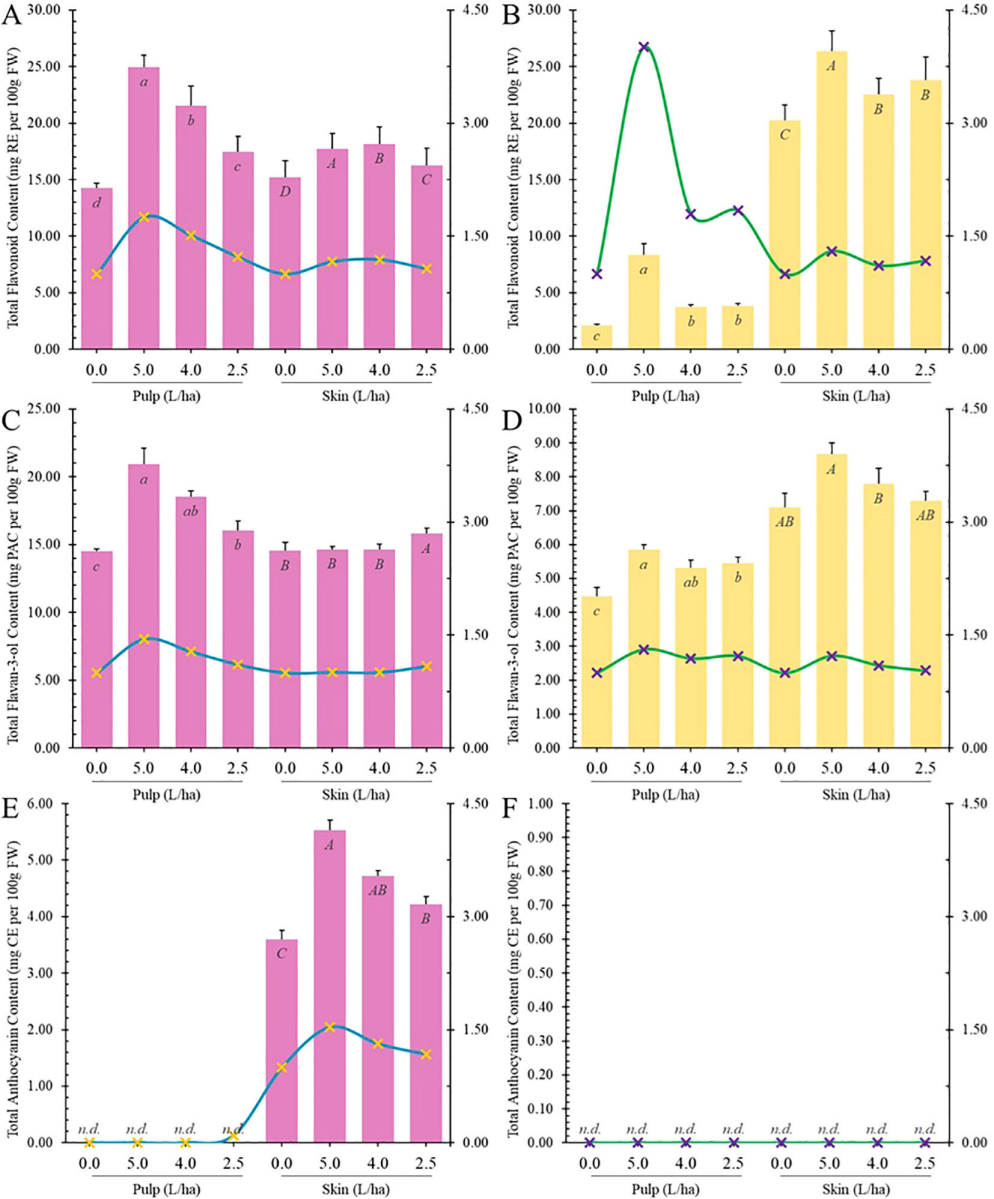
Total content of flavonoids **(A, B)**, flavan-3-ols **(C, D)** and anthocyanins **(E, F)** measured in the pulp or skin of Lady cot or Orange prima apricot fruit harvested from trees treated with different dosages of biostimulants (5.0 L/ha, 4.0 L/ha or 2.5 L/ha) or with water alone (0.0). Values are represented as mg ± SD per 100 g of FW. For each panel, within the same variety (Lady cot or Orange prima) different lowercase (pulp) or uppercase (skin) letters indicate significant differences at p ≤ 0.05, as measured by Tukey’s multiple interval test. The blue line represents the ratio between the values recorded in the respective treatments and the values of the control group, following the y-axis values indicated on the right. Numerical data are reported in **Supplementary Tables S4**, **S5**.

TPC is influenced by various phenolic classes and subclasses such as phenolic acids, flavonoids, and others. Spectrophotometric assays were employed to investigate if the potential impact of biostimulant application on specific classes of bioactive compounds. In our study, treatment with the biostimulant significantly increased total flavonoid content (TFC) in apricot fruits, both in pulp and peel, leading to statistically significant increases (p ≤ 0.05) (**Figures 6A, B**). For instance, in Lady cot cv., the 5.0 L/ha treatment increased TFC levels in peel by 1.16 folds and in pulp by 1.75 folds compared to the control. Similarly, the same treatment increased TFC levels in Orange prima pulp by 4.00 folds and in peel by 1.80 folds. The lowest biostimulant concentration treatment (2.5 L/ha) increased TFC in Lady cot cv. by 1.22 folds in pulp and by 1.07 folds in peel. In Orange prima cv., this treatment resulted in a 1.83-fold increase in pulp and a 1.17-fold increase in peel. Flavonoids showed significantly higher increases in the pulp of both cultivars compared to peel. Biostimulant treatment notably increased TFC in cv. Orange prima (white pulp) by 300% (TRT 5L/ha), 78.95% (TRT 4L/ha), and 83.73% (TRT 2.5L/ha). Dose-dependent correlations were observed in all treatments of cv. Lady cot, while in cv. Orange prima, while dose-dependent correlations were absent in 4.0 L/ha and 2.5 L/ha treatments (**Figures 6A, B**).

The discrepancies observed between TPC and TFC data may be attributed to the independent regulatory mechanisms of their respective biosynthetic pathways. While TPC includes a wider range of phenolic compounds, TFC specifically focuses on flavonoids, which can lead to variations in their levels following biostimulant treatments. However, it is essential to acknowledge the limitations inherent in colorimetric assays for measuring TPC and TFC, as these methods are often prone to errors and lack specificity. Therefore, the observed discrepancies between TPC and TFC data should be interpreted with caution, as variations in their measurements may not solely reflect differences in the underlying biosynthetic pathways but could also result from the inherent limitations of the colorimetric methods employed (Pisani et al., 2021).

Moreover, TFC is generally influenced by other flavonoid subclasses such as flavan-3-ols (TF3C) and anthocyanins (TAC). Proanthocyanidins (PACs) are oligomers or polymers of flavan-3-ol units, which belong to the polyphenol group and flavonoid sub-group, often known as condensed tannins (Mannino et al., 2021; Rauf et al., 2019). Dietary PACs with a degree of polymerization >3 are believed to remain unabsorbed in the gastrointestinal (GI) tract and accumulate in colon. PACs are also able to form non-specific complexes with salivary proteins in mouth, leading to the sensation of astringency, and with dietary proteins, pancreatic enzymes, and nutrient transporters in the intestinal lumen, thereby decreasing the digestion and absorption of carbohydrates, proteins, and lipids. Interestingly, PACs exhibit prebiotic activities by promoting the growth of *Lactobacillus* spp. and *Bifidobacterium* spp. as well as some butyrate-producing bacteria in the colon (Cires et al., 2017). PACs rich foods can promote intestinal health not only only through their antioxidant activity but also due the capacity of these phytochemicals to interact with multiple biomolecules, including proteins, lipids, and endotoxins (González-Quilen et al., 2020). Proanthocyanidins are also involved in the amelioration or prevention of age-dependent chronic diseases (Nie and Stürzenbaum, 2019) and by protecting the nervous system from oxidative stress damage (Gao et al., 2020). In our experimental conditions, untreated pulps of Lady cot variety averaged 14.49 ± 0.19 mg PACE/100 g FW (**Figure 6C**), while in Orange prima variety, it was 4.48 ± 0.25 mg PACE/100 g FW (**Figure 6D**). Untreated fruit peels of Lady cot variety showed an average of 14.54 ± 0.61 mg PACE per 100 g FW, and in Orange prima variety, it was 7.10 ± 0.41 mg PACE per 100 g FW (**Figures 6C, D**). Lady cot fruits exhibited similar proanthocyanidins (PACs) values in both pulp and peel, whereas in Orange prima variety, PACs were significantly higher in peel than in pulp. Despite the distance among genera, our findings align with previous research indicating an increase in TF3C levels in “Jonathan” apples (*Malus × domestica* Borkh) after treatment with a biostimulant based on a commercial seaweed extract (Algavis) (Soppelsa et al., 2020). Additionally, the application of a high chitosan dose increased the total flavan-3-ols at the middle veraison stage in white-colored cultivar Savvatiano grapes (Miliordos et al., 2022).

Anthocyanins, which are water-soluble polyphenols commonly known as pigments, are responsible for red, purple, and blue colors in fruits and vegetables. Cyanidin-3-hexoside isomer is the predominant anthocyanin found in many plants. The color and stability of anthocyanins are influenced by factors such as pH, light, and temperature. In acidic conditions, anthocyanins appear red but turn blue as pH increases (basic conditions) (Khoo et al., 2017). Preclinical, *in vitro*, *in vivo*, and clinical studies highlights the positive effects of anthocyanins in preventing various diseases across multiple systems, including the visual system (glaucoma, retinopathy, myopia), endocrine system (diabetes, obesity, hypercholesterolemia, hyperuricemia, thyroid, breast, and ovarian cancers), circulatory system (hypertension, heart disease, stroke), digestive system (nonalcoholic fatty liver disease, alcoholic fatty liver disease, gastric lesions, colorectal, liver, esophageal, oral, and pancreatic cancers), urinary system (renal injury, benign prostatic hyperplasia, prostate, and bladder cancers), nervous system (Alzheimer’s disease, Parkinson’s disease), and immune system (allergic and autoimmune diseases). Additionally, anthocyanins exhibit additional biological activities such as anti-infection (antibiosis, antiviral) and prevention against other cancers like lung and skin cancer (Liu et al., 2021). Under our experimental conditions, anthocyanins were detected only in the peel of cv. Lady cot (**Figures 6E, F**), and biostimulant treatments increased total anthocyanin content (TAC) in a dose-dependent manner. Specifically, the highest dosage resulted in improved anthocyanin content by about 50%, while other concentrations showed increases of approximately 30% and 15%, respectively (**Figure 6E**). Various studies have investigated the use of different typologies of biostimulants to enhance total anthocyanin content in fruits. For instance, in apples (Malus × domestica Borkh) of the cultivar ‘Jonathan’ grown in South Tyrol (Italy), various biostimulants, including humic acids, macro and micro seaweed extracts, alfalfa protein hydrolysate, amino acids, B-group vitamins, chitosan, and silicon-based products, were tested. All biostimulants led to an increase in total anthocyanin content, especially alfalfa protein hydrolysate, seaweed extracts, B-group vitamins, and chitosan, which doubled the anthocyanin content compared to the control (Soppelsa et al., 2018). Similarly, in ‘Jonathan’ apple trees, seaweed extracts significantly increased TAC levels (Soppelsa et al., 2018). In sweet cherry cv. ‘Staccato’, the application of glycine betaine-based biostimulants increased total anthocyanin levels Gonçalves et al., 2020). Trichoderma application resulted in a significant accumulation of total anthocyanins in strawberries (Lombardi et al., 2020). Moreover, the application of a biostimulant (SUNRED - 0.1% dilution) increased anthocyanin accumulation in ‘Red Globe’ grape peels (Deng et al., 2019).

### 3.4 Biostimulant treatment improved antioxidant properties of apricot fruits

Several scientific studies have demonstrated the capability of biostimulant treatment to increase antioxidant activity in fruits of different species, including apricot fruits (*Prunus armeniaca* L.) (Tarantino et al., 2018), apple fruits of Annurca cv (Graziani et al., 2020). and Clementine Mandarin (*Citrus clementina* Hort. Ex Tan) (Ziogas et al., 2022). Given the observed increase in total phenolic content (TPC) due to our biostimulant treatments, antioxidant activity assays have been performed to assess fruit antioxidant capacity after biostimulant treatments.

**Figure 7:**
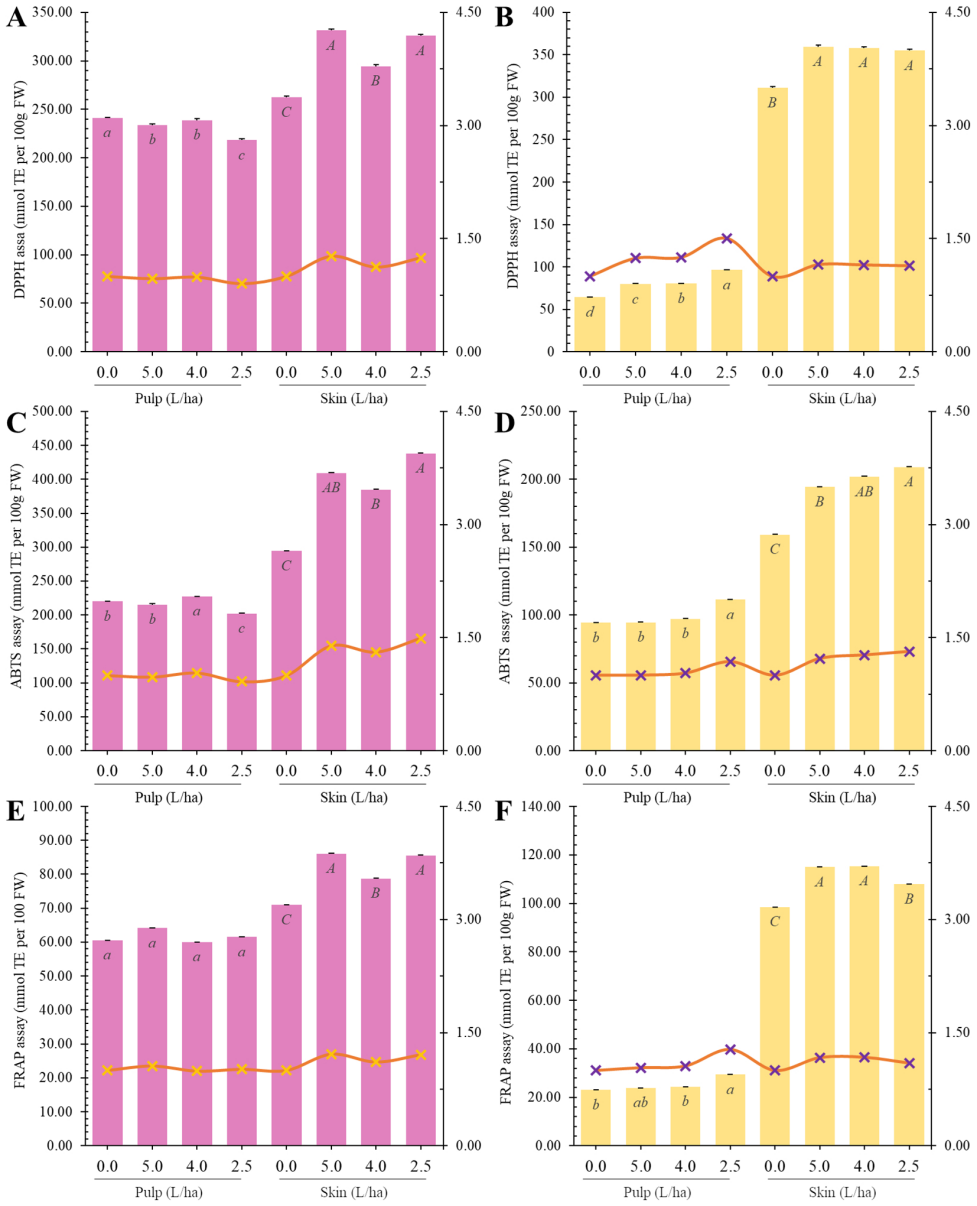
Radical Scavenging (DPPH: **(A, B)**; ABTS: **(C, D)** and Reducing (FRAP: **(E, F)** measured in the pulp or peel of Lady cot or Orange prima apricot fruit harvested from trees treated with different dosages of biostimulants (5.0 L/ha, 4.0 L/ha or 2.5 L/ha) or with water alone (0.0). Values are represented as mmol TE ± SD. For each panel, within the same variety (Lady cot or Orange prima) different lowercase letters indicate significant differences at p ≤ 0.05, as measured by Tukey’s multiple interval test. Orange and red line represent the ratio between the values recorded in the respective treatments and the values of the control group, following the y-axis values indicated on the right. Numerical data are reported in **Supplementary Tables S4**, **S5.** .

Under our experimental conditions, biostimulant treatments increased antioxidant capacity mainly in the peel of apricot fruits as measured by both ABTS and DPPH assays (**Figures 7A–D**).

Regarding DPPH assay results, the 5.0 L/ha biostimulant treatment decreased antioxidant capacity in Lady cot cv. pulp by 3.04% but increased it in the peel by 26.41%. In Orange prima cv., it increased antioxidant capacity in pulp by 19.31% and in peel by 15.61%. The 4.0 L/ha treatment left antioxidant capacity unchanged in Lady cot cv. pulps but increased it by +12.25% in peel, while in Orange prima cv., it increased by 26.22% in pulp and by 15.18% in peel. Conversely, the 2.5 L/ha treatment decreased antioxidant capacity of Lady cot apricots by 10.46% in pulp but increased it in peel by 24.26%. In Orange prima cv., it increased antioxidant capacity in pulp by 50.26% and in peel by 14.11% (**Figures 7A, B**).

In the ABTS assay, the 5.0 L/ha biostimulant treatment decreased antioxidant capacity in Lady cot cv. pulp by 2.13% but increased it in the peel by 39.12%. Similarly, it left antioxidant capacity unchanged in Orange prima cv. pulp but increased it in the peel by 22.20%. The 4.0 L/ha treatment increased antioxidant capacity in Lady cot cv. pulps by 3.33% and in peel by 30.84%, while in Orange prima cv., it increased by 2.88% in pulp and by 26.03% in peel. Conversely, the 2.5 L/ha treatment decreased antioxidant capacity of Lady cot apricots by 8.89% in pulp but increased it in peel by 48.97%. In Orange prima cv., it increased antioxidant capacity in pulp by 17.95% and in peel by 31.41% (**Figures 7C, D**).

Regarding the reducing activity, it was assessed via FRAP assay. Under our experimental conditions, biostimulant application mainly increased antioxidant activity in peels (**Figures 7E, F**). In Lady cot cv., treatment with 5.0 L/ha of biostimulant increased total antioxidant capacity of apricot pulps by 5.79% and peels by 21.34%. In Orange prima cv., the same treatment increased antioxidant capacity by 3.61% in pulps and by 16.80% in peels. The 4.0 L/ha biostimulant treatment left antioxidant capacity unchanged in pulps while increasing it in peels by 11.01% in Lady cot cultivar. In Orange prima cultivar, the same treatment increased antioxidant capacity by 5.91% in pulps and by 17.20% in peels. Finally, the smallest biostimulant treatment (2.5 L/ha) left antioxidant capacity unchanged in pulps while increasing it by 20.52% in peels of Lady cot cultivar. Regarding Orange prima cv., the same biostimulant treatment increased antioxidant capacity in pulps by 27.54% and in peels by 9.62% (**Figure 7F**).

Several studies have reported an increase in antioxidant capacity of fruits following treatment with one or more biostimulants. For instance, a single application of three different biostimulants (micro-algae, protein hydrolysate, and macro-algae combined with zinc and potassium) significantly increased the antioxidant capacity in the peel of Annurca cv. Apples. In contrast, the flesh antioxidant capacity was enhanced only by protein hydrolysate biostimulant (Graziani et al., 2020). Conversely, the application of the same biostimulant did not have a significant impact on the antioxidant capacity of *Solanum lycopersicum* L. var. Micro-Tom fruits (Mannino et al., 2020a).

## 4 Conclusion

In conclusion, our study highlights the multifaceted effects of biostimulant application on apricot fruit production, metabolism, and quality.

Although the treatments did not significantly affect the overall final fruit yield, they did impact maturation timing and the uniformity of harvestable fruits. This indicates a potential to reduce ripening time and achieve more consistent yields, both of which are crucial for enhancing harvesting efficiency and lowering labor costs. This could provide economic benefits to growers, particularly in scenarios where uniform fruit ripening is essential for mechanical harvesting, which in turn enhances profitability. Moreover, the increase in total phenolic and flavonoid content, as well as the improved antioxidant capacity observed in biostimulant-treated fruits, especially in the peel, offers potential post-harvest benefits. Higher antioxidant levels are known to be linked with extended shelf-life in soft fruits, which is an economic advantage for farmers and distributors by reducing spoilage and increasing the marketability of the product. Although we did not directly measure shelf-life improvements in this study, the observed phytochemical enhancements suggest this could be a promising area for further investigation, with clear financial implications. It is also worth considering the potential return on investment (RoI) and return on effort (RoE) for biostimulant treatments. While we did not perform an economic analysis in this study, the improved quality attributes of the fruits, such as enhanced nutritional value and shelf-life, are likely to translate into higher market prices and reduced post-harvest losses. Future research could explore these economic factors in more detail, evaluating the direct financial benefits to farmers from biostimulant applications. Key areas for future studies include optimizing biostimulant application protocols to maximize both economic returns and fruit quality, as well as investigating the direct impact of increased antioxidant content on shelf-life and post-harvest performance. By addressing these questions, we can better define the practical and financial benefits of biostimulant use in agricultural systems.

## Data Availability

The original contributions presented in the study are included in the article/**Supplementary Material**. Further inquiries can be directed to the corresponding author.
