# Supplementary material for: Seaweed and yeast extracts as sustainable phytostimulant to boost secondary metabolism of apricot fruits
Source: Front Plant Sci. 2025 Jan 24;15:1455156. doi: 10.3389/fpls.2024.1455156 (PMC11802282; doi:10.3389/fpls.2024.1455156)

Figure S1: Box plots and kernel density plots before and after normalization. The density plots are based on all samples. Selected methods : Row-wise normalization: by median; Data transformation: Log10 Normalization; Data scaling: Pareto Scaling.

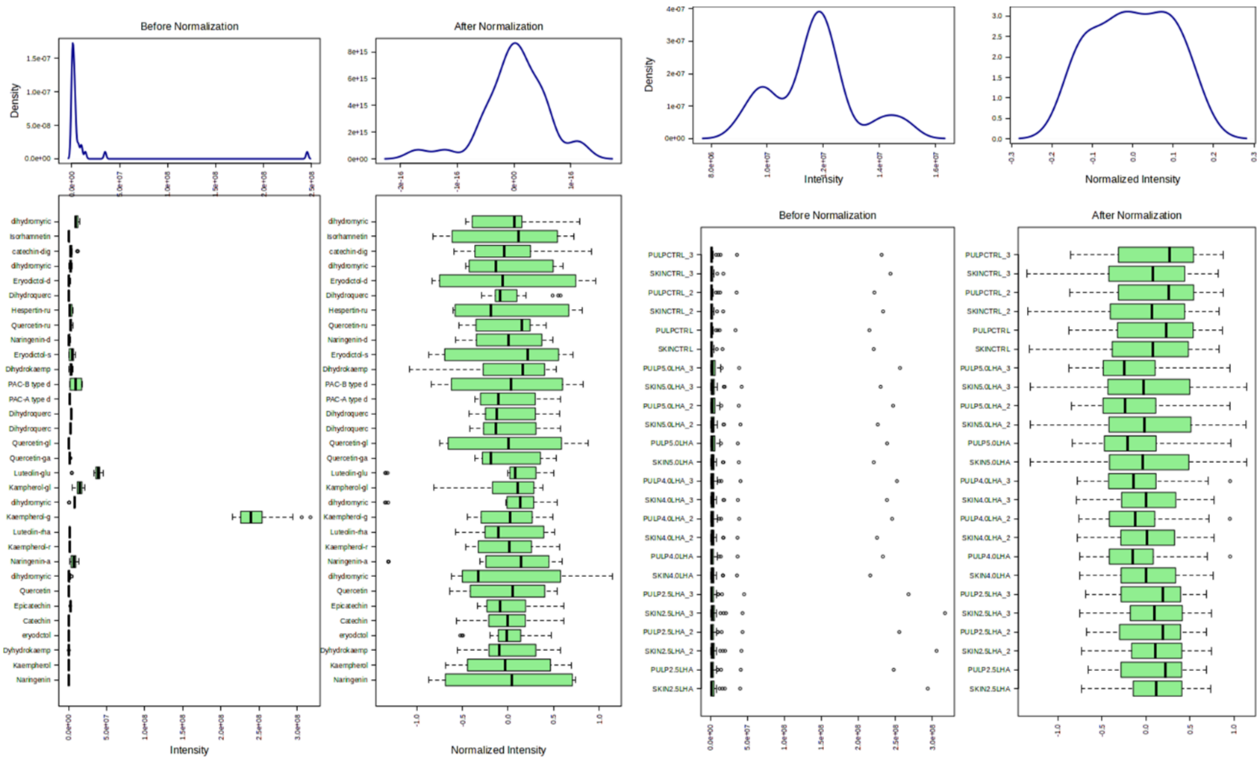

Supplement: Supplementary file 1 [file Image1.pdf]
