# Supplementary material for: Seaweed and yeast extracts as sustainable phytostimulant to boost secondary metabolism of apricot fruits
Source: Front Plant Sci. 2025 Jan 24;15:1455156. doi: 10.3389/fpls.2024.1455156 (PMC11802282; doi:10.3389/fpls.2024.1455156)

Figure S2: VIP score plot reporting the important features expressed as VIP scores, indicating the main variables that differentiate PULP from SKIN on Component 2, independently from the treatment application.

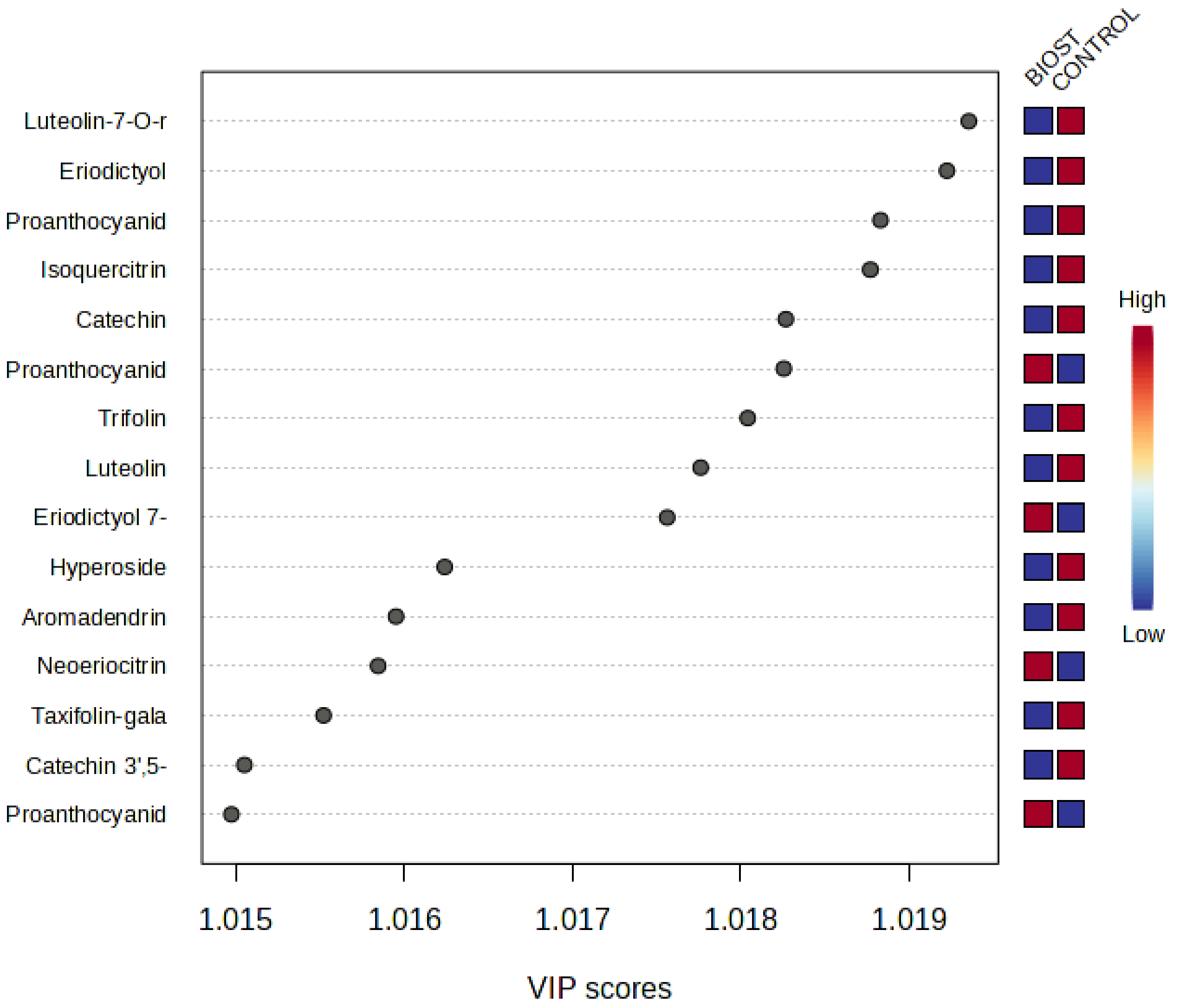

Supplement: Supplementary file 2 [file Image2.pdf]
