# Supplementary figures and images for: Seaweed and yeast extracts as sustainable phytostimulant to boost secondary metabolism of apricot fruits
Source: Front Plant Sci. 2025 Jan 24;15:1455156. doi: 10.3389/fpls.2024.1455156 (PMC11802282; doi:10.3389/fpls.2024.1455156)

**Figure S3:** Representative HPLC-MS/MS chromatogram of the compounds reported in Table S1.

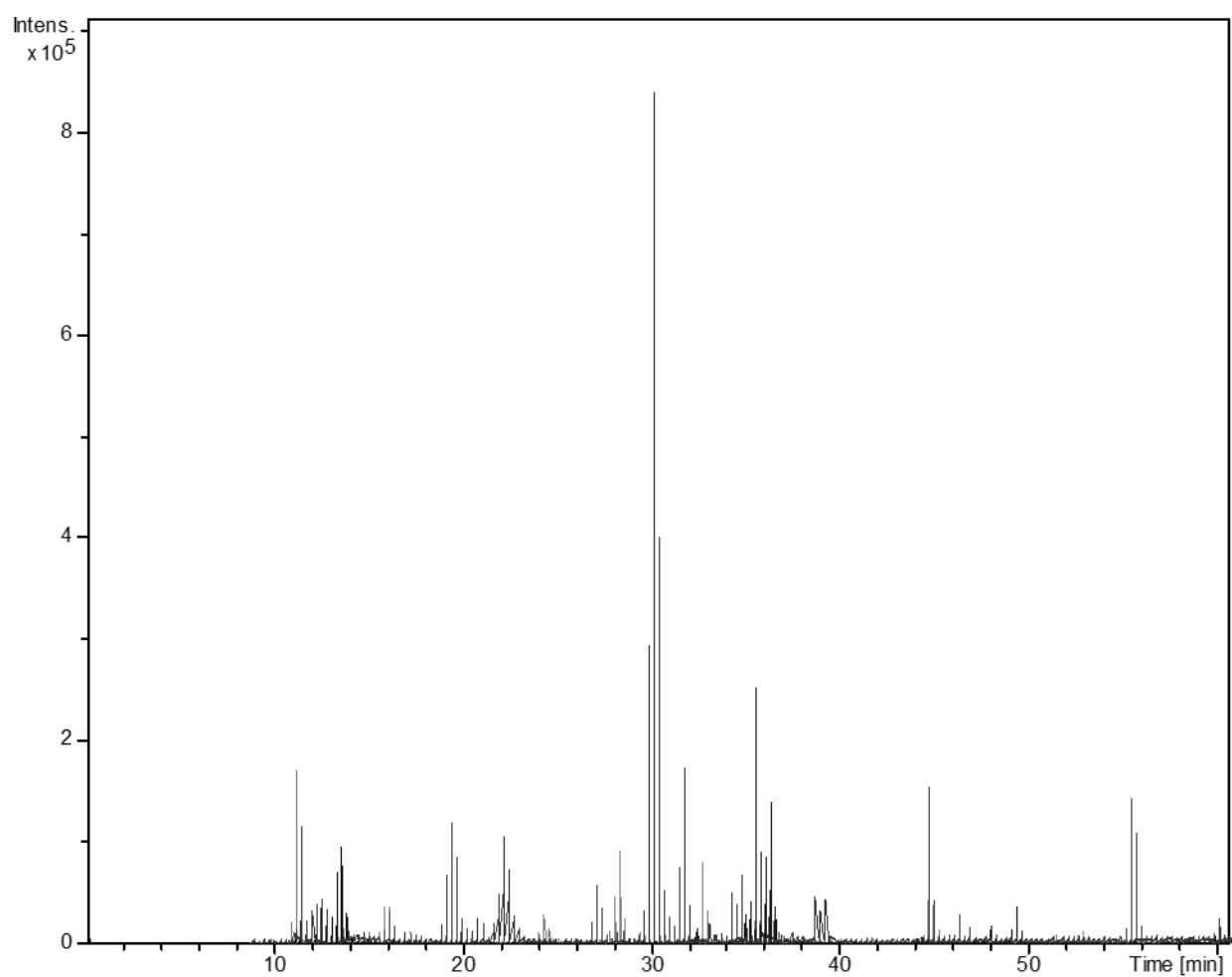

Supplement: Supplementary file 3 [file Image3.pdf]
