# Supplementary material for: Seaweed and yeast extracts as sustainable phytostimulant to boost secondary metabolism of apricot fruits
Source: Front Plant Sci. 2025 Jan 24;15:1455156. doi: 10.3389/fpls.2024.1455156 (PMC11802282; doi:10.3389/fpls.2024.1455156)
Supplement: Supplementary file 4 [file Table1.docx]

**Table S1**: Identification of bioactive compounds in skin or pulp of Orange Prima or Lady Cot varieties. Representative chromatogram of the selected compounds in shown in Figure S3.
