# Supplementary material for: Seaweed and yeast extracts as sustainable phytostimulant to boost secondary metabolism of apricot fruits
Source: Front Plant Sci. 2025 Jan 24;15:1455156. doi: 10.3389/fpls.2024.1455156 (PMC11802282; doi:10.3389/fpls.2024.1455156)

**Table S2**: Quantification of bioactive compounds in skin or pulp of Lady Cot var. via HPLC-DAD-MS/MS. Values refer to the amount (µg per 100 g of fresh weight) and are re expressed as mean ± SD of three different injection.


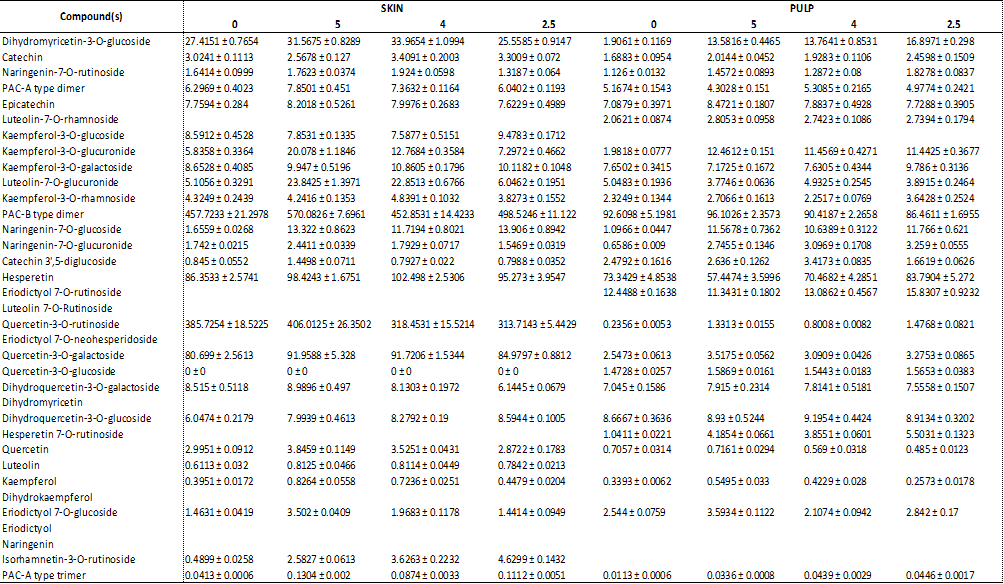

Supplement: Supplementary file 5 [file Table2.docx]
