# Supplementary material for: Seaweed and yeast extracts as sustainable phytostimulant to boost secondary metabolism of apricot fruits
Source: Front Plant Sci. 2025 Jan 24;15:1455156. doi: 10.3389/fpls.2024.1455156 (PMC11802282; doi:10.3389/fpls.2024.1455156)
Supplement: Supplementary file 8 [file Table5.docx]

**Table S5:** Quantification of bioactive compounds (TPC, TAC, TFC, and TF3C) and antioxidant potential (ABTS, DPPH, FRAP) in skin or pulp of Orange prima var. Values refer to the amount (µg per 100 g of fresh weight) and are re expressed as mean ± SD of three different injection.
